# Performance outcomes of the PEDI-CAT for assessing functional ability in the population with leukodystrophy

**DOI:** 10.1111/dmcn.70299

**Published:** 2026-04-30

**Authors:** Stacy V. Cusack, Allan M. Glanzman, Francesco Gavazzi, Sarah Woidill, Abbas F. Jawad, Timothy Estilow, Amy T. Waldman, Adeline Vanderver, Laura Adang, Children’s Hospital of Philadelphia Leukodystrophy Workgroup

**Affiliations:** 1 Department of Occupational Therapy, Children’s Hospital of Philadelphia, Philadelphia, PA, USA; 2 Department of Physical Therapy, Children’s Hospital of Philadelphia, Philadelphia, PA, USA; 3 Division of Neurology, Children’s Hospital of Philadelphia, Philadelphia, PA, USA; 4 Department of Neurology, Perelman School of Medicine, University of Pennsylvania, Philadelphia, PA, USA; 5 Division of General Pediatrics, Children’s Hospital of Philadelphia, Philadelphia, PA, USA; 6 Department of Pediatrics, University of Pennsylvania Perelman School of Medicine, Philadelphia, PA, USA

## Abstract

**Aim:**

To describe the use of the Pediatric Evaluation of Disability Index-Computer Adapted Test (PEDI-CAT), a parent-reported outcome, and determine functional performance in a cohort with leukodystrophy.

**Method:**

This was a cross-sectional observational study. Ninety-nine caregivers (of 46 females and 53 males, mean age = 7 years 11 months, age range = 2 months–34 years) completed the PEDI-CAT (Aicardi–Goutières syndrome *n* = 52, TUBB4A-related leukodystrophies *n* = 22, Pelizaeus–Merzbacher disease *n* = 7, POLR3-related leukodystrophy *n* = 18). Concurrent validity was compared between the PEDI-CAT mobility domain and the 88-item Gross Motor Function Measure (GMFM-88), and the PEDI-CAT daily activities domain with the visual motor integration (VMI) and grasping subtests of the Peabody Developmental Motor Scales, Second Edition (PDMS-2).

**Results:**

Strong correlations were observed between the PEDI-CAT mobility domain and the GMFM-88 (Spearman’s rank correlation coefficient: ρ = 0.93, 95% confidence interval [CI] = 0.90–0.95, *p* < 0.001), and the PEDI-CAT daily activities domain and the PDMS-2 VMI and grasping section (both Spearman’s rank correlation coefficient: ρ = 0.94, 95% CI = 0.91–0.96, *p* < 0.001).

**Interpretation:**

The PEDI-CAT is a simple, feasible tool for caregiver assessment of function in patients with leukodystrophies; a strong correlation with established assessment tools was achieved. These findings support the use of computerized adaptive tools as a faster way to stratify functional abilities in the population with leukodystrophy.

Leukodystrophies are a category of heterogeneous, inherited neurological disorders that affect the myelin of the central nervous system.^1,2^ Representing over 200 distinct monogenetic disorders, all leukodystrophies affect neurological function, although with variable severity and pace of progression. Clinical or functional trajectories can range from the relatively stable course of POLR3-related leukodystrophy (POLR3-LD) to variable courses seen in Aicardi–Goutières syndrome (AGS), TUBB4A-related leukodystrophy (TUBB4A-LD), and Pelizaeus–Merzbacher disease (PMD)^1–4^ Furthermore, depending on the specific disorder, age, and time from disease onset, children may have a broad range of function at the time of evaluation, which can make it difficult to anticipate needs and capture neurological function using a single clinical outcome assessment tool.

Performance outcomes are a subtype of clinical outcome assessments that are administered by trained professionals, compared to parent-reported outcome measures.^5,6^ Common performance outcomes used in the leukodystrophy space include the 88-item Gross Motor Function Measure (GMFM-88) and the Peabody Developmental Motor Scales, Second Edition (PDMS-2).^7–15^ The GMFM-88 captures five dimensions of gross motor function: lying and rolling; sitting; crawling and kneeling; standing; and walking, running, and jumping,^13^ and the PDMS-2 captures reflexes, stationary, locomotion, object manipulation, grasping, and visual motor integration (VMI).^7–9,15^ While these clinical outcome assessments reliably capture neurological function in the population with leukodystrophy, both provider-administered tools are time-intensive, require specialized training, and may overlook the parent or caregiver perspective on daily life accomplishments.^5,6^ Additionally, each performance outcome typically focuses on a specific subset of the severity spectrum, complicating the determination of which tools to use for assessing function given the evolving needs within neurodegenerative disorders.

A potential tool to address these limitations and provide complementary information on function is the Pediatric Evaluation of Disability Inventory-Computer Adaptive Test (PEDI-CAT), a parent-reported outcome measure.^16^ We have administered the PEDI-CAT as a parent or caregiver, or observer-reported, measure in alignment with US Food and Drug Administration guidelines.^5,6^ The PEDI-CAT has been validated across a range of neurodevelopmental disabilities, including spinal muscular atrophy, cerebral palsy, and autism spectrum disorder.^16–19^ As demonstrated by Fragala-Pinkham et al.,^17^ the PEDI-CAT can be used to assess daily function and mobility in spinal muscular atrophy types I to III, with good discriminant validity.^17^

The PEDI-CAT assesses function across four domains: daily activities; mobility; social/cognitive; and responsibility.^16–21^ The PEDI-CAT, which was first released in 2012, uses an algorithmic selection of questions from a bank (daily activities: 68 items; mobility: 75 items with an additional 12 if a walking aid is used; social/cognitive: 60 items; and responsibility: 51 items), which are specific to the patient based on prior answers.^16^ This approach optimizes administration efficiency and limits questions outside of a patient’s projected skill range.^16,20,21^

When administering the PEDI-CAT, the clinician can choose the speedy version (5–15 questions) or the content-balanced version (about 30 questions) for each domain.^16^ The potential advantages of the PEDI-CAT include brevity (fewer than 30 minutes to complete the content-balanced version), no set time between administrations, along with a wide age range and the capacity to capture the caregiver’s perspective on function.^16,20,21^ Additionally, its ability to now be remotely administered has the potential to reduce participation bias based on who can travel.^4,22^

Despite these strengths, the PEDI-CAT has yet to be explored in the population with leukodystrophy. Establishing its utility in this population is particularly relevant given disease heterogeneity, variable functional trajectories, and the logistical challenges associated with repeated in-person assessments.

In this article, we first aim to characterize the feasibility of administering the PEDI-CAT as a caregiver-reported measure of functional performance in a cohort of children with leukodystrophy. Second, we aim to evaluate convergent validity by examining associations between the PEDI-CAT mobility domain and the GMFM-88, and between the PEDI-CAT daily activities domain and the PDMS-2 grasping and VMI subtests. Lastly, we assess the use of the PEDI-CAT as a screening tool to triage patients with leukodystrophies for clinical and research purposes.

## METHOD

### Participants

Participants were recruited between 2017 and 2024 and provided written informed consent as per the institutional review board-approved (Children’s Hospital of Philadelphia institutional review board no. 14–011236) Myelin Disorders Biorepository Project. Research assessments were administered prospectively through November 2024. Inclusion criteria for the analysis included clinical and molecularly confirmed diagnosis of leukodystrophy and first concurrent administration of the PEDI-CAT, GMFM-88, and PDMS-2 in the context of research encounters at the Children’s Hospital of Philadelphia (*n* = 99; Table 1). Concurrent administration was defined as within 2 weeks (difference in date of administration: median = 0 days; minimum–maximum = 0–9 days).

### Study design

This was a cross-sectional observational study.

### Clinical outcome assessment selection and administration

The content-balanced PEDI-CAT (about 30 questions for each domain) and the PDMS-2 (VMI and grasping subtests) were administered by a trained occupational therapist. This therapist administered the PDMS-2, while a caregiver simultaneously completed the PEDI-CAT in the same session. The therapist was unaware of the score of the PEDI-CAT until the end of the session. Three parents completed the PEDI-CAT in their native language (Spanish). When the native language was not available, an in-person or telehealth interpreter was used to complete the assessment in English (*n* = 3). The GMFM-88 (all five dimensions) was completed by a trained physical therapist during their in-person research visit. Similarly, the physical therapist was not aware of the PEDI-CAT score.

The PEDI-CAT provides a standard opening question for each domain; subsequent questions (from the item bank) are adapted using item response theory. Caregivers choose from a 4-point or 5-point scale: easy; a little hard; hard; and unable. For all domains except the responsibility domain, there is an ‘I don’t know’ option. In the responsibility domain, options are as follows: the caregiver has full responsibility; the caregiver has most of the responsibility; the caregiver and child share responsibility roughly equally; the child has the most responsibility; and the child takes full responsibility. The PEDI-CAT generates an item map and assessment report with scaled scores, T-score, percentile, and fit. Although the recommended age range for its use is from birth up to 21 years old, scaled scores were used to include participants older than 21 years (*n* = 3), which is consistent with the manual guidance for skill-based longitudinal tracking.^16^

In addition, prior work has demonstrated alignment between the PEDI-CAT domains with the constructs from the International Classification of Functioning, Disability and Health for Children and Youth as described by Thompson et al. ^23–25^ Item-linking analyses have shown that 361 codes were associated with PEDI-CAT items; 99% map to the activities and participation category, with representation across all nine subchapters. Mobility (d4) accounted for the largest proportion of linked codes, followed by self-care (d5). The fewest links were observed in major life areas (d8). Also worth noting are two items in the responsibility domain that were ‘not definable’ using the International Classification of Functioning, Disability and Health codes.^25^

The PEDI-CAT also provides a fit score that represents the likelihood of answers given by the caregiver according to the item response theory software. Patterns of answers that cannot be predicted by the PEDI-CAT algorithm are labeled as ‘misfit’ if the fit score is more than 1.65 or less than −1.65. A subset of participants fell in the ‘misfit’ range but were still included in this paper. The planned time for administration of the assessment was 30 minutes. The reported average time for the full content-balanced PEDI-CAT is 12.66 minutes; the minimum was 3.96 minutes and the maximum was 26.68 minutes.^16,20,21^ The completion time was not collected in the context of this study.

### Content validity

Disease and outcome measures experts (SVC, LAA, AV, AG, FG) established content validity between the PEDI-CAT daily activities and PDMS-2 VMI and grasping subtests in the context of a focus group (unanimous agreement). The VMI consists of 72 items, while the grasping subtest includes 26 items, with scores ranging from 0 to 2 (VMI range 0–144 and grasping 0–52). Normative data are available for ages 0 to 71 months. As the PDMS-2 was administered outside the normative age range, only raw scores were considered in the context of this study. Floor effect was defined as raw scores less than 10% of the maximum possible score (VMI < 15 and grasping <6), while the ceiling effect was classified as scores greater than 90% (> 129 and grasping > 46) as described previously. ^15^ The same expert group (SVC, LAA, AV, AG, FG) established content validity between the PEDI-CAT mobility domain and the GMFM-88 (unanimous agreement). For the GMFM-88, each dimension’s scores are summarized to achieve a raw and a percentage score. Dimension percentage scores are averaged to obtain a total percentage score, considered in the context of statistical analysis.^13^ As published previously, the floor was defined as below 20% and the ceiling was above 20%.^3,11,12^

### Data management

The PEDI-CAT data were stored on a dedicated research iPad and transferred quarterly into a PostgreSQL table (PostgreSQL Global Development Group Regents of the University of California, Berkeley, CA, USA) and queried with REDCap (Vanderbilt University, Nashville, TN, USA) outcomes and demographic data, all saved on a secure drive. The GMFM-88 and PDMS-2 data were extracted from source documents (scoring booklets) and entered in REDCap. REDCap is a secure, web-based software platform designed to support data capture for research studies.^26–28^

### Statistical analysis

Descriptive statistics were generated for all variables. Continuous variables, including the mean, SD, median, interquartile range (IQR) (Q1–Q3), minimum and maximum, and coefficient of variation (CV), were generated for continuous variables and frequency percentages for categorical variables. Pairwise mean differences with 95% confidence intervals (CIs) were used to describe differences in scores across disorders (AGS, PMD, POLR3-LD, TUBB4A-LD). Spearman’s rank correlation was used to assess the association between the PEDI-CAT domain scores and the PDMS-2 VMI and grasping raw score and GMFM-88 total percentage score, provider-administered assessments for the cohort. Fisher’s z-transformation was used to compare correlation coefficients between acceptable and misfit groups. All analyses used two-sided testing and an alpha level of 0.05. All analyses and visualizations were performed in R v4.2.2 (R Foundation for Statistical Computing, Vienna, Austria).^28^

## RESULTS

### Cohort description

Ninety-nine individuals were enrolled in the study: 52 with AGS (52.5%), 22 with TUBB4A-LD (22.2%), seven with PMD (7.1%), and 18 with POLR3-LD (18.2%). Demographic information, including sex, age, ethnicity, and residence, and function (PEDI-CAT language and adaptive mobility) are summarized in Table 1. The cohort was evenly distributed according to sex, with 46.5% female and 53.5% male. The population was mostly white (*n* = 80, 80.7%) residing in the USA (*n* = 89, 89.9%; Table 1). The mean age at the time of administration was 7 years 11 months (SD = 6 years 5 months, median = 6 years 2 months, IQR = 2 years 11 months–11 years 11 months, age range = 2 months–34 years; Table 1). The widest distribution was found in the population with PMD (range = 2 months–34 years). POLR3-LD had the highest mean age of 12 years 7 months (range = 2 years 4 months–23 years 8 months, IQR = 7 years 5 months–18 years 7 months) as seen in Table 1 and Figure S1. Ninety-seven percent (*n* = 96) of PEDI-CAT assessments were completed in English with the remaining 3% (*n* = 3) completed using the Spanish version (Table 1). Regarding mobility aids, 20.2% of participants used a walker and 39.4% used a manual wheelchair, although only 19.2% independently propelled their manual wheelchair (Table 1). Power wheelchair use was reported in 9.1% (Table 1). Cane use was rarely reported (1.0%; Table 1).

### Clinical outcome assessment performance

**FIGURE 1:**
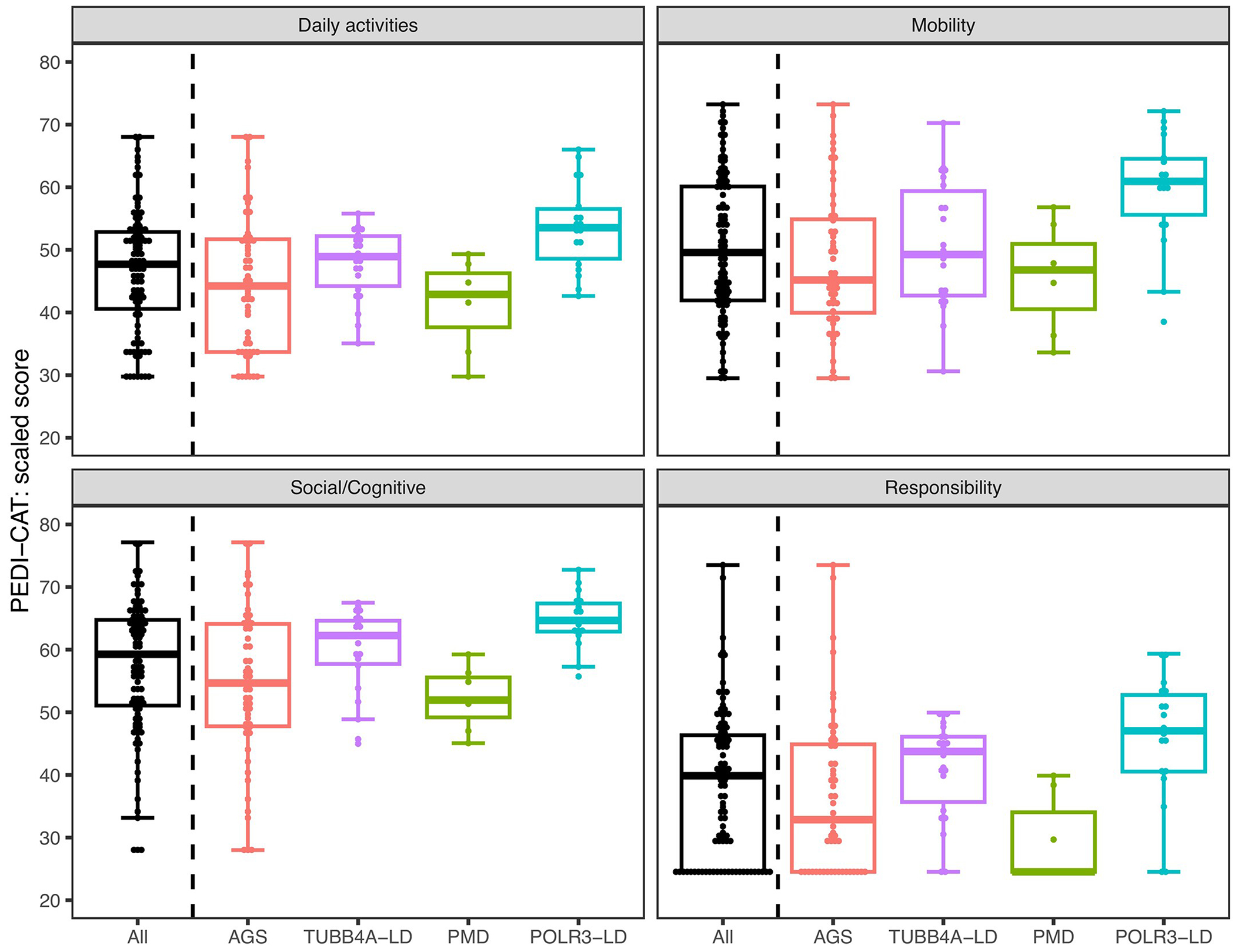
Distribution of Pediatric Evaluation of Disability Inventory-Computer Adaptive Test (PEDI-CAT) performance according to diagnosis. The PEDI-CAT was completed by 99 caregivers of individuals with leukodystrophy. The performance across the four domains, for the overall cohort versus the subcohorts of Aicardi–Goutières syndrome (AGS), Pelizaeus–Merzbacher disease (PMD), POLR3-related leukodystrophy (POLR3-LD), and TUBB4A-related leukodystrophy (TUBB4A-LD) was captured. As per Table 2: daily activities: mean = 46.7, SD = 9.56, median = 47.7 (IQR = 40.6–52.9); mobility: mean = 50.5, SD = 11.2, median = 49.6 (IQR = 40.9–60.1); social/cognitive, mean = 57.3, SD = 10.6, median = 59.3 (IQR = 51.1–64.8); responsibility: mean = 38.7, SD = 11.7, median = 49.9 (IQR = 24.5–46.3). See Table 4 for the distribution of subcohort scores.

PEDI-CAT performance across the PEDI-CAT domains is summarized in Table 2, Table S1, Figure 1, and Figure S2. PEDI-CAT domain scores are reported as scaled scores. PEDI-CAT domain scores demonstrated a wide range of functional performance (Table 2). Mean scaled scores were 46.7 (SD = 9.56) for daily activities, 50.5 (SD = 11.2) for mobility, 38.7 (SD = 11.7) for social/cognitive, and 57.3 (SD = 10.6) for responsibility, with similar median scores. The highest relative variability in PEDI-CAT scaled score was observed in the responsibility domain (CV = 30.3%).

**FIGURE 2:**
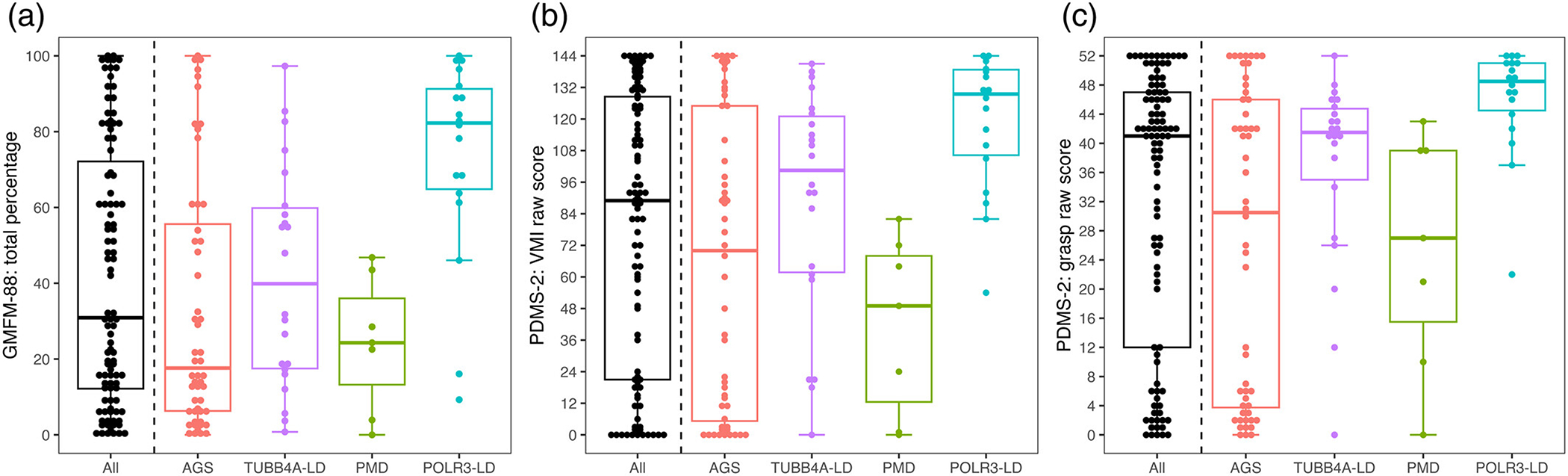
Cohort scores on performance outcomes. (a) GMFM-88 total percentage, mean = 41.8, SD = 33.9, median = 30.9 (IQR = 12.2–72.1). (b) PDMS-2 VMI raw score, mean = 78.6, SD = 52.2, median = 89.0 (IQR = 21.0–129.0). (c) PDMS-2 grasping raw score, mean = 32.2, SD = 18.6, median = 41.0 (IQR = 12.0–47.0). See Table 4 for the distribution of subcohort scores. Abbreviations: AGS, Aicardi–Goutières syndrome; GMFM-88, 88-item Gross Motor Function Measure; PDMS-2, Peabody Developmental Motor Scales, Second Edition; PMD, Pelizaeus–Merzbacher disease; POLR3-LD, POLR3-related leukodystrophy; TUBB4A-LD, TUBB4A-related leukodystrophy; VMI, visual motor integration.

The clinical severity spectrum across diagnoses was captured through performance-based outcome measures in Table 2 and Figure 2. The GMFM-88 total score ranged from 0% to 100% (mean = 41.8, SD = 33.9, median = 30.9, IQR = 12.2–72.1). Fine motor performance was assessed using the PDMS-2. The PDMS-2 VMI raw scores ranged from 0 to 144 (mean = 78.6, SD = 52.2, median = 89.0, IQR = 21.0–129); grasping raw scores ranged from 0 to 52 (mean = 32.2, SD = 18.6, median = 41.0, IQR = 12.0–47.0).

Strong associations were observed among PEDI-CAT domains and performance-based outcome measures (Table 3). Correlations across PEDI-CAT domains ranged from 0.74 to 0.90 (Table 3). The PEDI-CAT mobility domain demonstrated a strong correlation with the GMFM-88 total score (ρ = 0.93, 95% CI = 0.90–0.95, *p <* 0.001), which is consistent with both measures assessing gross motor function. Similarly, the PEDI-CAT daily activities scores were strongly associated with the PDMS-2 fine motor outcomes, including VMI (ρ = 0.94, 95% CI = 0.91–0.96, *p* < 0.001) and grasping (ρ = 0.94, 95% CI = 0.91–0.96, *p* < 0.001).

Descriptive statistics stratified according to diagnosis are presented in Table 4. Variability in functional performance was observed across subgroups. Participants with POLR3-LD (*n* = 18) demonstrated significantly higher mean scores in the mobility domain and GMFM-88 when compared with other disorders (mean difference in mobility and GMFM-88 respectively: AGS-POLR3-LD, *n* = 70: −11.94 [95% CI = −19.38 to −4.49] and − 40.5 [95% CI = −62.28 to −18.7]; PMD-POLR3-LD, *n* = 25: −14.05 [95% CI = −26.182 to −1.93] and − 49.36 [95% CI = −84.84 to −13.9]; POLR3-LD-TUBB4A-LD, *n* = 40: 9.28 [95% CI = 0.62–17.93] and 31.63 [95% CI = 6.32–56.9]; Table 5). This group also showed higher mean gross and fine motor performance, as well as all other PEDI-CAT domain scores, when compared with AGS and PMD (Table 5). Fewer significant differences in performance were observed between AGS, TUBB4A-LD, and PMD (Table 5).

In contrast, individuals with AGS (*n* = 52) exhibited the widest distribution of functional abilities, reflected by large CVs across PEDI-CAT domains (daily activities, 24.0%; mobility, 23.3%; social/cognitive, 22.9%; responsibility, 34.9%) and GMFM-88 (100%) and PDMS-2 measures (VMI, 85.7%; grasping, 79.2%; Table 4). The mean GMFM-88 total score in the group with AGS was 33.1% (SD = 33.2, median = 17.6, IQR = 6.30–55.6, range = 0–100; Table 4). Participants with TUBB4A-LD (*n* = 22) demonstrated intermediate functional performance across domains with less variability (daily activities, mean = 47.9, SD = 5.60, CV% = 11.7; mobility, mean = 50.5 SD = 10.2, CV% = 20.2; social/cognitive, mean = 59.8, SD = 6.77, CV% = 11.3; responsibility, mean = 41.0, SD = 7.78, CV% = 18.0; Table 4 and Figure 1).

Low mean scores were observed in the subgroup with PMD across PEDI-CAT domains (daily activities, mean = 41.4, SD = 7.20, CV% = 17.4; mobility, mean = 45.7, SD = 8.50, CV% = 18.6; social/cognitive, mean = 52.3, SD = 5.03, CV% = 9.6; responsibility, mean = 29.4, SD = 6.90, CV% = 23.4; Table 4 and Figure 1) and performance-based measures (GMFM-88, mean = 24.2, SD = 17.8, CV% = 73.5; PDMS-2 VMI, mean = 41.7, SD = 33.7, CV% = 80.7; PDMS-2 grasping, mean = 25.6, SD = 16.2, CV% = 63.5; Table 4); however, interpretation is limited by the small sample size (*n* = 7).

**FIGURE 3:**
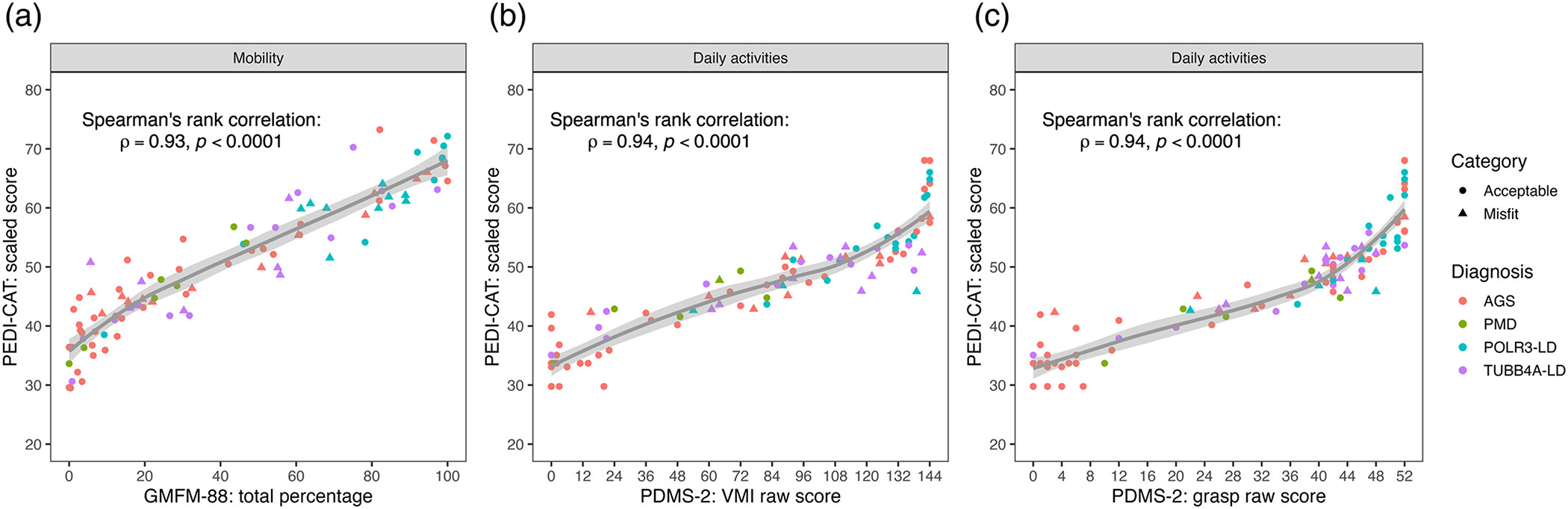
Association of Pediatric Evaluation of Disability Inventory-Computer Adaptive Test (PEDI-CAT) performance and clinical outcome assessments. All 99 participants completed concurrent 88-item Gross Motor Function Measure (GMFM-88) and Peabody Developmental Motor Scales, Second Edition (PDMS-2) assessments within 2 weeks of PEDI-CAT administration. Locally estimated scatterplot smoothing trend lines are shown in gray. (a) The total GMFM-88 percentage was strongly and significantly correlated with the PEDI-CAT mobility domain scaled scores (Spearman’s rank correlation: ρ = 0.93, *p* < 0.001, 95% CI = 0.90–0.95). (b) Performance in the PDMS-2 visual motor integration (VMI) was strongly and significantly correlated with the PEDI-CAT daily activities domain scaled scores (Spearman’s rank correlation: ρ = 0.94, *p* < 0.001, 95% CI = 0.91–0.96). (c) Performance in PDMS-2 grasping was strongly and significantly correlated with the PEDI-CAT daily activities domain scaled scores (Spearman’s rank correlation: ρ = 0.94, *p* < 0.001, 95% CI = 0.91–0.96). Abbreviations: AGS, Aicardi–Goutières syndrome; PMD, Pelizaeus–Merzbacher disease; POLR3-LD, POLR3-related leukodystrophy; TUBB4A-LD, TUBB4A-related leukodystrophy.

Table 5 demonstrates statistically significant differences with POLR3-LD, individuals, and both AGS and PMD across part-reported and performance-based outcome measures. AGS demonstrated significantly lower scores across all PEDI-CAT domains, including daily activities, mobility, social/cognitive, and responsibility, with mean differences ranging from −9.13 to −11.94 points. Participants with AGS also showed substantially lower gross and fine motor performance relative to POLR3-LD, including lower GMFM-88 total scores (mean difference = −40.5%) and lower PDMS-2 VMI and grasping scores. Similarly, participants with PMD demonstrated significantly lower functional performance compared with POLR3-LD across PEDI-CAT domains and performance-based measures. Differences were particularly pronounced for the mobility, social/cognitive, and responsibility domains, as well as GMFM-88 total scores and PDMS-2 VMI scores. Accepted and misfit were explored in Figure 3, Figure S3, and Table 1.

## DISCUSSION

The broad and evolving clinical spectrum of leukodystrophies complicates anticipating the acquisition of skills; therefore, this affects the selection of the appropriate outcome measures. This study characterized the use of the PEDI-CAT to capture functional performance across several leukodystrophies, with a wide range of abilities and ages. The PEDI-CAT highlights the use of a parent-reported measure to complement performance outcomes and address the gaps in assessments available for rare neurogenerative disorders.

The PEDI-CAT demonstrated a strong correlation with the GMFM-88 and PDMS-2. This study confirmed the established association between the PEDI-CAT mobility domain and the GMFM-88 and further validated the daily activities domain by comparing it with the PDMS-2-administered measures of fine motor skills, specifically the VMI and grasping subsections. Interestingly, strong correlations were also observed between the PEDI-CAT responsibility and social/cognitive domains and the PDMS-2 scores (range = 0.86–0.91). Associations between the PEDI-CAT domains and the GMFM-88 ranged from 0.76 to 0.93, reflecting expected construct alignment across measures of daily functioning and mobility.

Furthermore, our findings support the PEDI-CAT’s conceptual alignment with the activities and participation category of the International Classification of Functioning, Disability and Health for Children and Youth, an established international classification system, as demonstrated in Thompson et al.,^25^ supporting the content validity of the PEDI-CAT with a focus on function. Other International Classification of Functioning, Disability and Health areas like body function, body structure, and environmental factors are underrepresented in the PEDI-CAT items as they are naturally impeded in the activities and participation category. ^23–25^

The impact of functional abilities reported in each domain differed across diagnoses, supporting a diverse phenotypic spectrum within this class of rare disorders. Although AGS was the largest disease cohort, functional abilities varied widely across individuals. This wide range highlights the heterogeneity of AGS and emphasizes the importance of a measure that can capture both low-functioning and higher-functioning individuals within the same disease cohort. Patients with POLR3-LD demonstrated higher functional scores compared with other diagnoses across all domains, while patients with PMD demonstrated the lowest scores in the responsibility domain. However, the responsibility domain is intended to assess 3-year-old children and does not offer a ‘I don’t know’ option.^16^ While we had all parents complete this domain regardless of their child’s age, it is important to consider that most patients at the floor level are appropriately less than 3 years old (Figure S2). Further studies within disease-specific cohorts will help to understand the PEDI-CAT in disease subcohort stratification. Importantly, the PEDI-CAT adds value by capturing the caregiver’s perspective, an essential component recently recognized in the literature.^29,30^

The PEDI-CAT uses an adapted item response theory algorithm to determine which questions are asked next based on previous answers. The fit score indicates how answers align with the predicted response of the algorithm. Approximately one quarter of the administrations in the population with leukodystrophy were classified as ‘misfits’ (most notably in the responsibility domain and among older patients). The distinction between fit and misfit did not seem to affect overall performance. We hypothesized that the misfit scores arise from atypical patterns of developmental gains and losses within this unique population over time. We anticipated that patients with limited motor function would have misfit scores; however, most misfit patients had a scaled score of 35 and greater, therefore they were not the patients who had a floor effect.

Future studies may explore the potential of the PEDI-CAT for screening individuals for eligibility in clinical trials and for longitudinal remote monitoring after intervention. The use of an online, web-based assessment like the PEDI-CAT enables the long-term follow-up of patients across geographical regions and preferred languages.

### Limitations

The current study included only the English and Spanish versions of the PEDI-CAT, although a broader range of languages is currently available. This study was limited to in-person administration to allow comparison with standard outcome measures. Despite the small sample size in certain populations because of rare diseases, we demonstrated strong, significant associations, attesting to the robustness of the relationship between the administered and parent-reported measures. The use of the web-based version of the PEDI-CAT could broaden the reach and generalizability of our preliminary findings. Future studies could include a comparison between the speedy version of the PEDI-CAT and the content-balanced version within populations with leukodystrophy. Another potential limitation of this study is that the responsibility domain is intended to assess children aged 3 years and older. In this study, all sections were completed regardless of age. Notably, most patients at the floor level for this domain were appropriately aged less than 3 years old.

## Conclusion

These results support the utility of the PEDI-CAT as an efficient caregiver-centered tool that aligns with provider-administered assessments and captures the functional diversity seen in leukodystrophies. Its implementation could facilitate more inclusive outcome measurement in both clinical and research settings. The PEDI-CAT is a straight-forward severity stratification tool that can offer valuable insights into the perspectives of patients and caregivers regarding function. This was a cross-sectional study of the use of the PEDI-CAT with the population with leukodystrophies; future longitudinal studies should look at meaningful clinical relevance, along with capturing change of function over time.

## Supplementary Material

Figure 1 Supplemental The following additional material may be found online:
**Figure S1.** PEDI-CAT distribution of age at assessment by disorder.

Figure 2 Supplemental **Figure S2.** PEDI-CAT scaled scores by age at assessment.

Figure 3 Supplemental **Figure S3.** Correlation scores according to fit score.

Table 1 Supplemental **Table S1.** Domain comparison according to fit score.

## Figures and Tables

**TABLE 1:** Cohort description (*n* = 99).

Characteristic	*n* (%)
Sex	
Female	46 (46.5)
Male	53 (53.5)

Diagnosis	
AGS	52 (52.5)
TUBB4A-LD	22 (22.2)
PMD	7 (7.1)
POLR3-LD	18 (18.2)

Age (years:months)	
Mean (SD)	7:11 (6:5)
Median (Q1–Q3)	6:2 (2:11–11:11)
Minimum, maximum	0:2, 34:0

Ethnicity	
Biracial/multiracial	7 (7.1)
Black/African American	3 (3.0)
White	80 (80.8)
Hispanic/Latino	2 (2.0)
Other	5 (5.1)
Unknown/not reported	2 (2.0)

Residence	
International	10 (10.1)
USA	89 (89.9)

PEDI-CAT language	
English	96 (97.0)
Spanish	3 (3.0)

Uses walker	
No	79 (79.8)
Yes	20 (20.2)

Uses cane	
No	98 (99.0)
Yes	1 (1.0)

Uses manual wheelchair	
No	60 (60.6)
Yes	39 (39.4)

Propels manual wheelchair	
No	80 (80.8)
Yes	19 (19.2)

Uses power wheelchair	
No	90 (90.9)
Yes	9 (9.1)

Abbreviations: AGS, Aicardi–Goutières syndrome; PEDI-CAT, Pediatric Evaluation of Disability Inventory-Computer Adaptive Test; PMD, Pelizaeus–Merzbacher disease; POLR3-LD, POLR3-related leukodystrophy; TUBB4A-LD, TUBB4A-related leukodystrophy.

*Notes*: Data are shown as *n* (%) unless stated otherwise.

**TABLE 2:** Descriptive statistics for functional outcome measures.

Score	*n*	Mean (SD)	Median (Q1–Q3)	Minimum, maximum	CV%
PEDI-CAT (scaled score)					
Daily activities score (20–80)	99	46.7 (9.56)	47.7 (40.6–52.9)	29.8, 68.0	20.5
Mobility score (20–80)	99	50.5 (11.2)	49.6 (41.9–60.1)	29.5, 73.2	22.3
Social/cognitive score (20–80)	99	57.3 (10.6)	59.3 (51.1–64.8)	28.0, 77.1	18.6
Responsibility score (20–80)	99	38.7 (11.7)	39.9 (24.5–46.3)	24.5, 73.5	30.3

GMFM-88					
Total % (0–100)	99	41.8 (33.9)	30.9 (12.2–72.1)	0, 100	81.0

PDMS-2					
VMI raw score (0–144)	99	78.6 (52.2)	89.0 (21.0–129)	0, 144	66.3
Grasping raw score (0–52)	99	32.2 (18.6)	41.0 (12.0–47.0)	0, 52.0	57.9

Abbreviations: GMFM-88, 88-item Gross Motor Function Measure; PDMS-2, Peabody Developmental Motor Scales, Second Edition; PEDI-CAT, Pediatric Evaluation of Disability Inventory-Computer Adaptive Test; VMI, visual motor integration.

*Notes*: The PEDI-CAT, GMFM-88, and PDMS-2 scores have been summarized using the mean (SD), median (Q1–Q3), range, and coefficient of variation percentage (CV%).

**TABLE 3:** PEDI-CAT domains and performance-based outcome measures.

Score	PEDI-CAT: daily activities score	PEDI-CAT: mobility score	PEDI-CAT: social/cognitive score	PEDI-CAT: responsibility score	GMFM-88: total (%)	PDMS-2: VMI score (raw)
PEDI-CAT: daily activities score	1.00	0.89	0.87	0.87	0.90	**0.94**
PEDI-CAT: mobility score	0.89	1.00	0.74	0.74	**0.93**	0.82
PEDI-CAT: responsibility score	0.87	0.74	0.90	0.90	0.78	0.87
PEDI-CAT: social/cognitive score	0.87	0.74	1.00	1.00	0.76	0.91
GMFM-88: total (%)	0.90	**0.93**	0.76	0.78	1.00	0.88
PDMS-2: VMI score (raw)	**0.94**	0.82	0.91	0.87	0.88	1.00
PDMS-2: grasping score (raw)	**0.94**	0.83	0.88	0.86	0.87	0.96

Abbreviations: GMFM-88, 88-item Gross Motor Function Measure; PDMS-2, Peabody Developmental Motor Scales, Second Edition; PEDI-CAT, Pediatric Evaluation of Disability Inventory-Computer Adaptive Test; VMI, visual motor integration.

*Notes*: Bold type indicates the correlations of interest between the PEDI-CAT domain and GMFM-88 or PDMS-2. Spearman’s and Pearson’s rank correlation coefficients have been used to describe associations between PEDI-CAT scaled score, GMFM-88 total score, and PDMS-2 VMI and grasping raw scores.

**TABLE 4:** Functional outcomes according to diagnosis.

Group	Variable	*n*	Mean (SD)	Median (Q1–Q3)	Minimum, maximum	CV%
AGS	Age (years:months)	52	6:5 (5:4)	4:9 (2:6–8:5)	0:2, 22:8	84.0
	PEDI-CAT: daily activities score	52	44.5 (10.7)	44.2 (33.7–51.7)	29.8, 68.0	24.0
	PEDI-CAT: mobility score	52	47.9 (11.1)	45.2 (40.0–54.9)	29.5, 73.2	23.3
	PEDI-CAT: social/cognitive score	52	54.3 (12.4)	54.7 (47.8–64.1)	28.0, 77.1	22.9
	PEDI-CAT: responsibility score	52	36.5 (12.7)	32.8 (24.5–44.9)	24.5, 73.5	34.9
	GMFM-88: total (%)	52	33.1 (33.2)	17.6 (6.30–55.6)	0, 100	100.4
	PDMS-2: VMI score (raw)	52	65.0 (55.7)	70.0 (5.25–125)	0, 144	85.7
	PDMS-2: grasping score (raw)	52	26.1 (20.7)	30.5 (3.75–46.0)	0, 52.0	79.2

TUBB4A-LD	Age (years:months)	22	8:10 (4:7)	7:8 (4:5–12:6)	1:2, 18:6	56.6
	PEDI-CAT: daily activities score	22	47.9 (5.60)	48.9 (44.2–52.2)	35.1, 55.8	11.7
	PEDI-CAT: mobility score	22	50.5 (10.2)	49.3 (42.7–59.4)	30.6, 70.3	20.2
	PEDI-CAT: social/cognitive score	22	59.8 (6.77)	62.3 (57.7–64.6)	45.0, 67.5	11.3
	PEDI-CAT: responsibility score	22	41.0 (7.78)	43.7 (35.7–46.1)	24.5, 50.0	18.9
	GMFM-88: total (%)	22	42.0 (29.1)	39.9 (17.5–59.8)	0.780, 97.3	69.4
	PDMS-2: VMI score (raw)	22	89.2 (43.0)	101 (61.8–121)	0, 141	48.2
	PDMS-2: grasping score (raw)	22	37.2 (12.7)	41.5 (35.0–44.8)	0, 52.0	34.3

PMD	Age (years:months)	7	6:11 (12:0)	2:1 (1:9–3:7)	1:5, 34:0	174.2
	PEDI-CAT: daily activities score	7	41.4 (7.20)	42.9 (37.6–46.3)	29.8, 49.3	17.4
	PEDI-CAT: mobility score	7	45.7 (8.50)	46.8 (40.5–51.0)	33.6, 56.8	18.6
	PEDI-CAT: social/cognitive score	7	52.3 (5.03)	52.0 (49.2–55.6)	45.1, 59.2	9.6
	PEDI-CAT: responsibility score	7	29.4 (6.90)	24.5 (24.5–34.0)	24.5, 39.9	23.4
	GMFM-88: total (%)	7	24.2 (17.8)	24.3 (13.2–36.0)	0, 46.8	73.5
	PDMS-2: VMI score (raw)	7	41.7 (33.7)	49.0 (12.5–68.0)	0, 82.0	80.7
	PDMS-2: grasping score (raw)	7	25.6 (16.2)	27.0 (15.5–39.0)	0, 43.0	63.5

POLR3-LD	Age (years:months)	18	12:7 (6:5)	12:1 (7:6–18:7)	2:4, 23:8	51.1
	PEDI-CAT: daily activities score	18	53.6 (6.89)	53.5 (48.6–56.5)	42.6, 66.0	12.9
	PEDI-CAT: mobility score	18	59.8 (8.91)	60.9 (55.6–64.5)	38.5, 72.1	14.9
	PEDI-CAT: social/cognitive score	18	64.8 (4.30)	64.7 (62.9–67.4)	55.7, 72.8	6.6
	PEDI-CAT: responsibility score	18	45.6 (10.1)	47.0 (40.5–52.8)	24.5, 59.3	22.2
	GMFM-88: total (%)	18	73.6 (26.7)	82.3 (64.8–91.3)	9.28, 100	36.3
	PDMS-2: VMI score (raw)	18	119 (25.9)	130 (106–139)	54.0, 144	21.7
	PDMS-2: grasping score (raw)	18	46.1 (7.42)	48.5 (44.5–51.0)	22.0, 52.0	16.1

Abbreviations: AGS, Aicardi–Goutières syndrome; GMFM-88, 88-item Gross Motor Function Measure; PDMS-2, Peabody Developmental Motor Scales, Second Edition; PEDI-CAT, Pediatric Evaluation of Disability Inventory-Computer Adaptive Test; PMD, Pelizaeus–Merzbacher disease; POLR3-LD, POLR3-related leukodystrophy; TUBB4A-LD, TUBB4A-related leukodystrophy; VMI, visual motor integration.

*Notes*: Age and functional outcome measures have been summarized for each diagnostic group using the mean (SD), median (Q1–Q3), range, and coefficient of variation percentage (CV%).

**TABLE 5:** Diagnosis-based comparisons across functional outcome measures.

Score	AGS-PMD (*n* = 59), mean estimate (95% CI)	AGS-POLR3-LD (*n* = 70), mean estimate (95% CI)	AGS-TUBB4A-LD (*n* = 74), mean estimate (95% CI)	PMD-POLR3-LD (*n* = 25), mean estimate (95% CI)	PMD-TUBB4A-LD (*n* = 29), mean estimate (95% CI)	POLR3-LD-TUBB4A-LD (*n* = 40), mean estimate (95% CI)
PEDI-CAT: daily activities score	3.07 (−6.35 to 12.48)	**−9.18 (−15.58 to −2.79)**	−3.44 (−9.38 to 2.51)	**−12.25 (−22.66 to −1.83)**	−6.5 (−16.65 to 3.64)	5.74 (−1.69 to 13.18)
PEDI-CAT: mobility score	2.12 (−8.845 to 13.08)	**−11.94 (−19.383 to −4.49)**	−2.66 (−9.586 to 4.26)	**−14.05 (−26.182 to −1.93)**	−4.78 (−16.593 to 7.04)	**9.28 (0.622–17.93)**
PEDI-CAT: social/cognitive score	2.03 (−8.38 to 12.44)	**−10.51 (−17.58 to −3.44)**	−5.53 (−12.11 to 1.04)	**−12.53 (−24.05 to −1.02)**	−7.56 (−18.78 to 3.66)	4.97 (−3.25 to 13.19)
PEDI-CAT: responsibility score	7.04 (−4.58 to 18.651)	**−9.13 (−17.02 to −1.243)**	−4.56 (−11.9 to 2.773)	**−16.17 (−29.02 to −3.318)**	−11.6 (−24.12 to 0.919)	4.57 (−4.6 to 13.738)
GMFM-88: total (%)	8.86 (−23.2 to 40.9)	**−40.5 (−62.28 to −18.7)**	−8.87 (−29.12 to 11.4)	**−49.36 (−84.84 to −13.9)**	−17.73 (−52.29 to 16.8)	**31.63 (6.32–56.9)**
PDMS-2: VMI score (raw)	23.3 (−26.81 to 73.42)	**−54.3 (−88.3 to −20.22)**	−24.2 (−55.82 to 7.49)	**−77.6 (−133.01 to −22.12)**	−47.5 (−101.48 to 6.54)	30.1 (−9.46 to 69.65)
PDMS-2: grasping score (raw)	0.563 (−17.44 to 18.569)	**−19.976 (−32.21 to −7.746)**	−11.047 (−22.42 to 0.327)	**−20.54 (−40.46 to −0.619)**	−11.61 (−31.02 to 7.797)	8.929 (−5.28 to 23.143)

Abbreviations: GMFM-88, 88-item Gross Motor Function Measure; PDMS-2, Peabody Developmental Motor Scales, Second Edition; PEDI-CAT, Pediatric Evaluation of Disability Inventory-Computer Adaptive Test; VMI, visual motor integration.

*Notes*: Bold type indicates a 95% confidence interval (CI) that does not include 0 indicating the comparison/values are significantly different. Adjusted mean differences (95% CIs) are shown for pairwise comparisons between Aicardi–Goutières syndrome (AGS), TUBB4A-related leukodystrophy (TUBB4A-LD), Pelizaeus–Merzbacher disease (PMD), and POLR3-related leukodystrophy (POLR3-LD). Values represent the mean score of the first diagnosis minus the second diagnosis listed. Negative values indicate lower performance in the first diagnosis relative to the comparator. Comparisons with 95% CIs that do not cross zero indicate statistically significant differences after age adjustment.

## Data Availability

Data available on request from the authors.

## What this paper adds

- The Pediatric Evaluation of Disability Index-Computer Adapted Test (PEDI-CAT) captures skills in patients with leukodystrophy.
- The PEDI-CAT correlates with widely accepted performance outcome tools.
- The PEDI-CAT can help capture function before formal assessments.
- The PEDI-CAT can be used as a disease triage tool.

## Abbreviations

AGS: Aicardi–Goutières syndrome
GMFM-88: 88-item Gross Motor Function Measure
PDMS-2: Peabody Developmental Motor Scales, Second Edition
PEDI-CAT: Pediatric Evaluation of Disability Index-Computer Adapted Test
PMD: Pelizaeus–Merzbacher disease
POLR3-LD: POLR3-related leukodystrophy
TUBB4A-LD: TUBB4A-related leukodystrophy
VMI: visual motor integration
