# Supplementary figures and images for: Performance outcomes of the PEDI-CAT for assessing functional ability in the population with leukodystrophy
Source: Dev Med Child Neurol. Author manuscript; Available in PMC 2026 Jul 19. (PMC13380813; doi:10.1111/dmcn.70299)

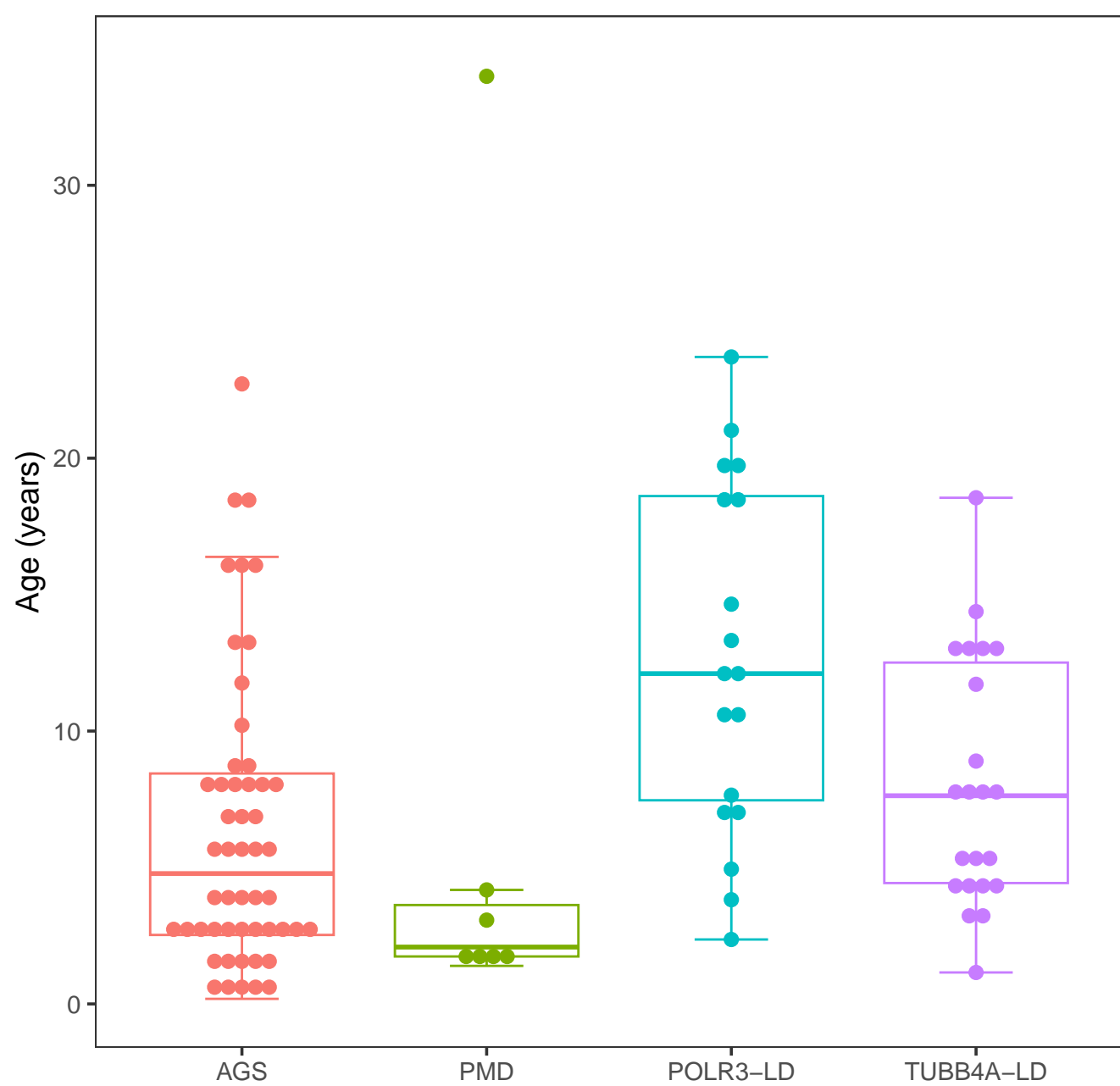

Supplement: Figure 1 Supplemental — The following additional material may be found online: Figure S1. PEDI-CAT distribution of age at assessment by disorder. [file NIHMS2190353-supplement-Figure_1_Supplemental.pdf]

# PEDI-CAT Scaled Scores by Age

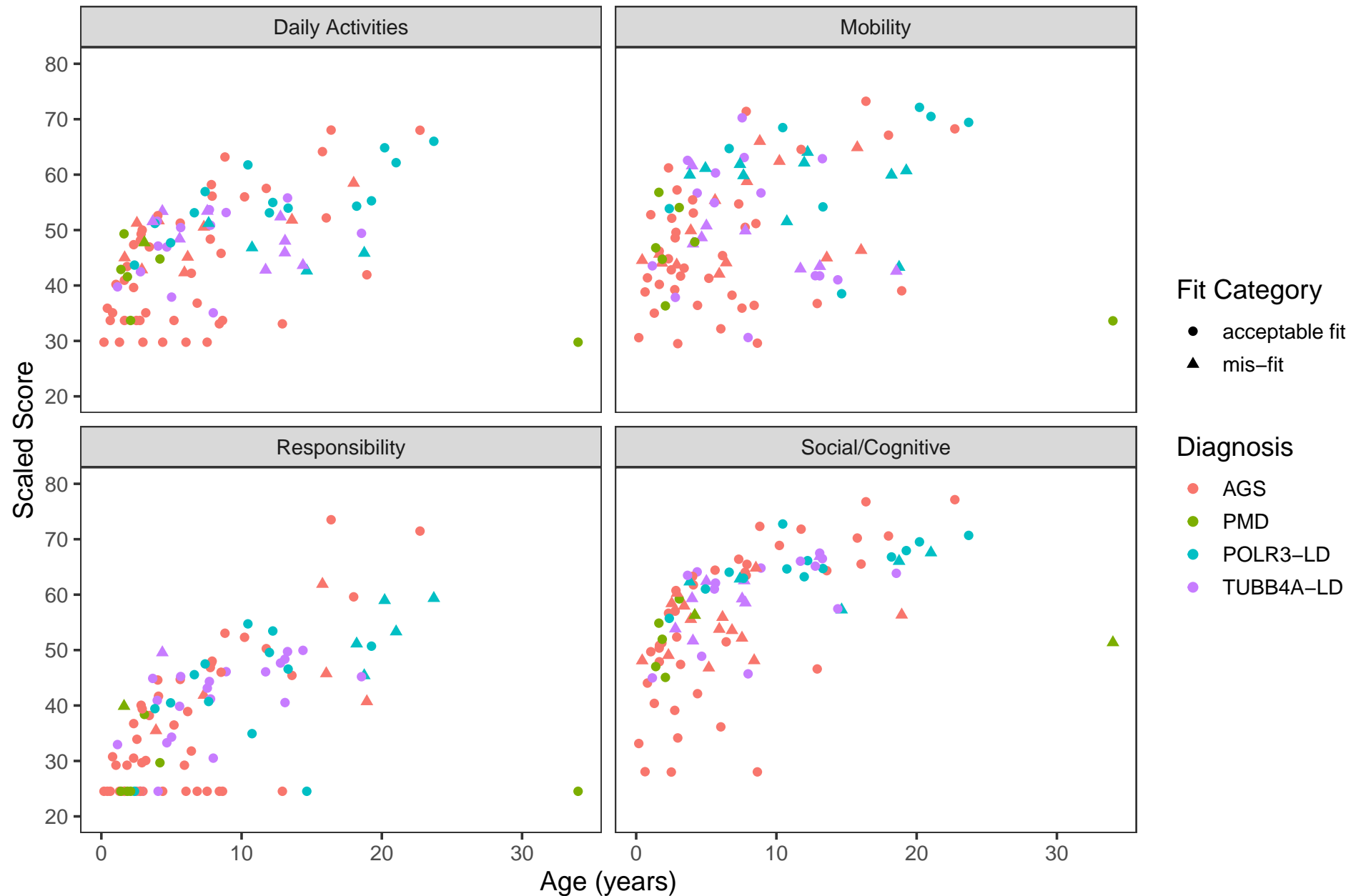

Supplement: Figure 2 Supplemental — Figure S2. PEDI-CAT scaled scores by age at assessment. [file NIHMS2190353-supplement-Figure_2_Supplemental.pdf]

**(a)**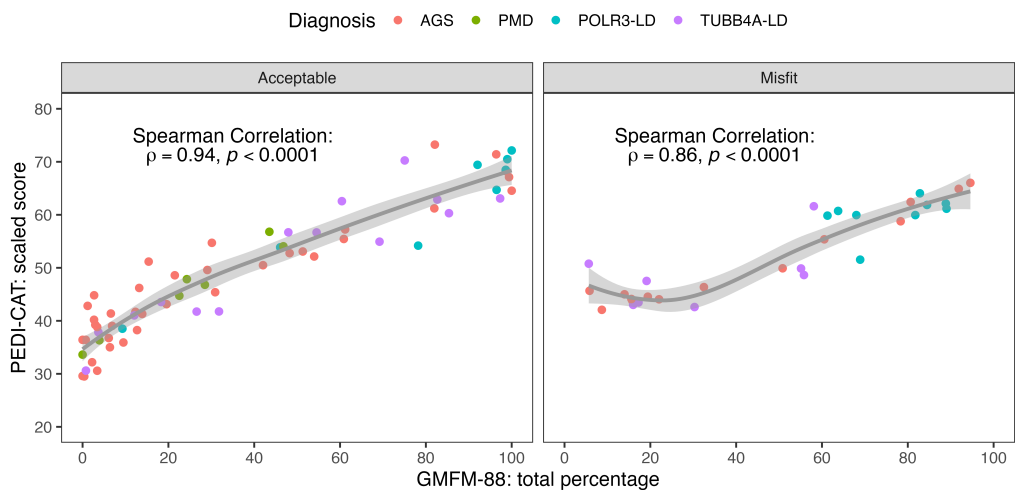**(b)**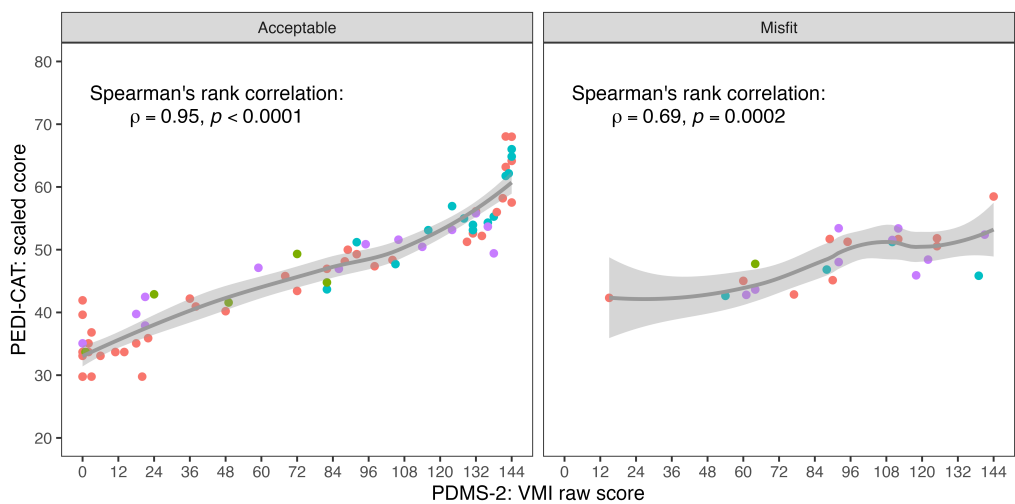**(c)**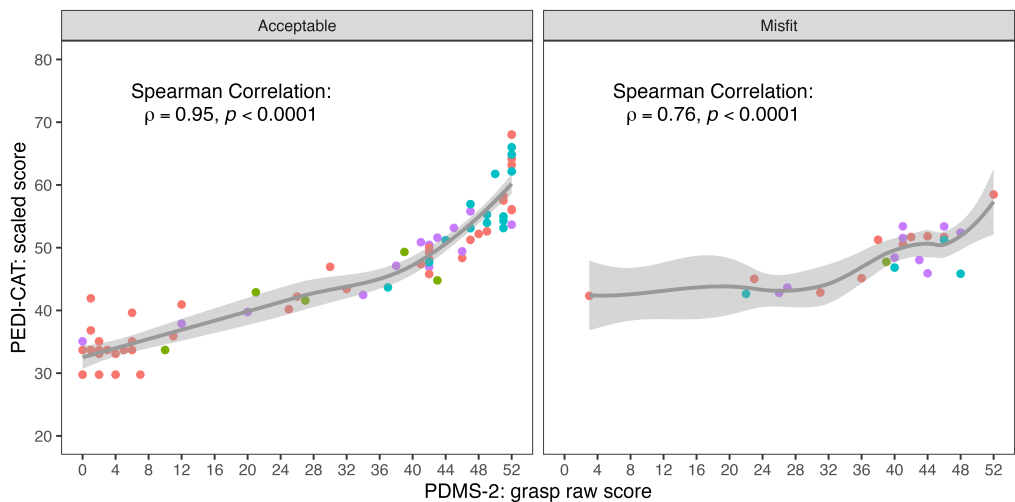

Supplement: Figure 3 Supplemental — Figure S3. Correlation scores according to fit score. [file NIHMS2190353-supplement-Figure_3_Supplemental.pdf]
