# Supplementary material for: Performance outcomes of the PEDI-CAT for assessing functional ability in the population with leukodystrophy
Source: Dev Med Child Neurol. Author manuscript; Available in PMC 2026 Jul 19. (PMC13380813; doi:10.1111/dmcn.70299)
Supplement: Table 1 Supplemental — Table S1. Domain comparison according to fit score. [file NIHMS2190353-supplement-Table_1_Supplemental.docx]

**Table S1. Domain comparison by fit score**

|  | **Daily Activities** | | | **Mobility** | | |
| --- | --- | --- | --- | --- | --- | --- |
|  | **acceptable fit** | **mis-fit** | **P-value** | **acceptable fit** | **mis-fit** | **P-value** |
| N (%) | 75 (75.8%) | 24 (24.2%) |  | 67 (67.7%) | 32 (32.3%) |  |
| Age (years) |  |  | 0.2176 |  |  | 0.0826 |
| Median (IQR) | 5.66 (7.57) | 7.43 (9.06) |  | 5.58 (7.03) | 7.72 (7.93) |  |
| [Min, Max] | [0.186, 34.0] | [1.66, 18.7] |  | [0.186, 34.0] | [0.428, 19.3] |  |
| [Q1, Q3] | [2.77, 10.3] | [4.03, 13.1] |  | [2.64, 9.67] | [4.51, 12.4] |  |
| Scaled Score |  |  | 0.3034 |  |  | 0.1061 |
| Median (IQR) | 47.1 (17.9) | 48.2 (6.58) |  | 47.9 (17.9) | 50.3 (16.4) |  |
| [Min, Max] | [29.8, 68.0] | [42.3, 58.5] |  | [29.5, 73.2] | [42.1, 66.0] |  |
| [Q1, Q3] | [35.5, 53.4] | [45.1, 51.7] |  | [39.1, 57.0] | [44.4, 60.8] |  |
| Diagnosis |  |  | 0.2162 |  |  | 0.0345 |
| AGS | 42 (56.0%) | 10 (41.7%) |  | 38 (56.7%) | 14 (43.8%) |  |
| TUBB4A-LD | 13 (17.3%) | 9 (37.5%) |  | 14 (20.9%) | 8 (25.0%) |  |
| PMD | 6 (8.0%) | 1 (4.2%) |  | 7 (10.4%) | 0 (0%) |  |
| POLR3-LD | 14 (18.7%) | 4 (16.7%) |  | 8 (11.9%) | 10 (31.3%) |  |
|  | **Social/Cognitive** | | | **Responsibility** | | |
|  | **acceptable fit** | **mis-fit** | **P-value** | **acceptable fit** | **mis-fit** | **P-value** |
| N (%) | 70 (70.7%) | 29 (29.3%) |  | 87 (87.9%) | 12 (12.1%) |  |
| Age (years) |  |  | 0.7468 |  |  | 0.0035 |
| Median (IQR) | 6.23 (9.41) | 6.16 (4.52) |  | 5.66 (6.74) | 17.1 (12.7) |  |
| [Min, Max] | [0.186, 23.7] | [0.428, 34.0] |  | [0.186, 34.0] | [1.62, 23.7] |  |
| [Q1, Q3] | [2.76, 12.2] | [3.89, 8.41] |  | [2.81, 9.56] | [6.57, 19.3] |  |
| Scaled Score |  |  | 0.3522 |  |  | 0.0014 |
| Median (IQR) | 61.9 (16.4) | 57.3 (8.77) |  | 38.2 (21.3) | 47.7 (13.1) |  |
| [Min, Max] | [28.0, 77.1] | [46.8, 67.6] |  | [24.5, 73.5] | [35.5, 61.9] |  |
| [Q1, Q3] | [49.1, 65.5] | [53.6, 62.3] |  | [24.5, 45.8] | [41.6, 54.7] |  |
| Diagnosis |  |  | 0.8747 |  |  | 0.1291 |
| AGS | 38 (54.3%) | 14 (48.3%) |  | 47 (54.0%) | 5 (41.7%) |  |
| TUBB4A-LD | 14 (20.0%) | 8 (27.6%) |  | 21 (24.1%) | 1 (8.3%) |  |
| PMD | 5 (7.1%) | 2 (6.9%) |  | 6 (6.9%) | 1 (8.3%) |  |
| POLR3-LD | 13 (18.6%) | 5 (17.2%) |  | 13 (14.9%) | 5 (41.7%) |  |
